# Your move: A precision medicine framework for physical activity in aging

**DOI:** 10.1038/s41514-024-00141-9

**Published:** 2024-02-27

**Authors:** Adrián Noriega de la Colina, Timothy P. Morris, Arthur F. Kramer, Navin Kaushal, Maiya R. Geddes

**Affiliations:** 1 The Montreal Neurological Institute-Hospital, McGill University, 3801 Rue University, Montréal, QC Canada; 2 Department of Neurology and Neurosurgery, Faculty of Medicine and Human Sciences, McGill University, 3801 Rue University, Montréal, QC Canada; 3 Department of Physical Therapy, Movement and Rehabilitation Sciences, Northeastern University, Boston, USA; 4 Center for Cognitive and Brain Health, Northeastern University, Boston, USA; 5 School of Health & Human Sciences, Indiana University, Indiana, USA

**Keywords:** Risk factors, Cardiovascular diseases, Neurodegeneration, Ageing

## Abstract

The accelerating digital health landscape, coupled with the proliferation of wearable devices and advanced neuroimaging, offers an unprecedented avenue to develop precision interventions for enhancing physical activity in aging. This approach requires deep baseline phenotyping to match older adults with the intervention poised to yield maximal health benefits. However, building sufficient evidence to translate precision physical activity recommendations into clinical practice requires a collaborative effort that includes accessible open data. We propose a strategic roadmap to design and implement personalized programs, effectively decreasing physical inactivity and bolstering adherence among older adults.

Physical inactivity is a significant global health challenge and a leading risk factor for chronic illnesses and premature death^1^. An estimated 27.5% of the world’s population, or 1.4 billion adults, do not engage in sufficient physical activity^1^. This prevalence varies between high- and low-income countries (36.8% vs 16.2%, respectively), as well as between men and women (23.4% vs 31.7%)^1^. Physical inactivity also increases as the population ages, ranging from 59.8% in middle-aged adults 45–64 years old to 65.7% in older adults 65 and older in the United States^2^. Despite efforts from multiple public health agencies worldwide, the percentage of physically inactive adults has increased since 1995^3^.

The World Health Organization (WHO) set a goal of reducing physical inactivity by 15% worldwide by 2030 through its Global Action Plan on Physical Activity 2018–2030^4^. Compounding this problem, the COVID-19 pandemic has disrupted trends in physical activity engagement worldwide, and we are still far from achieving the 2030 goals, especially for older adults who significantly reduced their physical activity levels during the pandemic. The WHO recommends that all adults aged 18 years and older engage in 150–300 minutes of moderate-intensity or 75–150 minutes of vigorous-intensity physical activity per week^5^. Nevertheless, the effectiveness of widespread initiatives targeting physical activity promotion at the population level has been limited, resulting in only marginal reported improvements while facing the difficulty of maintaining behavior change over the long term^6^. A study of the US National Health and Nutrition Examination Surveys found no improvement in adherence to physical activity guidelines from 2007–2008 to 2015–2016, and during the same period, there was an increase in sedentary time^7^. With the current trend, the WHO’s 2030 objective of reducing physical inactivity by 15% worldwide is unlikely to be met. Moreover, while other countries report higher levels of physical activity engagement than the US, there is still a large portion of their populations that are not meeting the recommended thresholds^6^. This is especially true for individuals with chronic conditions such as cancer, cardiovascular disease and diabetes^8^, who represent half of all older adults. Among older adults with more severe chronic diseases, setting the same recommended weekly guidelines as the general older adult population has not always translated into effective adherence. The low adherence to physical activity guidelines among individuals with chronic diseases can be partially attributed to mobility limitations and a higher prevalence of low socioeconomic status among these populations^9^. Therefore, it is important to consider whether individuals with chronic conditions face unique barriers to adhering to physical activity targets.

To optimize the health benefits of physical activity, it is crucial to consider variations in Minimal Clinically Important Differences (MCID) and tailor activity goals accordingly. A meta-analysis investigating the relationship between physical activity and cognitive function in older adults with a healthy body mass index has identified diverse dose-response ranges for different types of activities. Among these, aerobic and resistance exercises demonstrated an inverted U-shaped pattern with optimal benefits at an intermediate level^10^. This can be attributed to the distinct mechanisms through which various exercise modalities impact health outcomes, as well as variances in perceived fatigue and effort^10^. Interestingly, the study found that overweight and obese individuals experienced cognitive benefits at lower activity levels than the general population guidelines recommended by the WHO. These effects were larger in older women as compared to older men, without reporting for race. It is worth noting that a meta-analysis of physical activity interventions suggested modest group-level effect sizes in cognitive outcomes, and that some effects may become smaller when accounting for certain moderators like for example baseline performance or training program duration^11^. However, it is central to recognize that this meta-analysis presented many methodological issues, the most relevant being that it excluded individuals with multiple medical conditions, leading to biased sample selection^11^. Therefore, to ensure that physical activity is optimized for health benefits, it is important to acknowledge that populations with comorbidities may present variations in MCID, which emphasizes the necessity of tailoring physical activity goals to the individual.

When analyzing complex comorbid conditions such as neurodegenerative disease, dose-response ranges are less well understood. Research on physical activity interventions for patients with Alzheimer’s disease (AD) and related disorders has produced mixed findings regarding the optimal intervention duration for achieving positive health outcomes. One meta-analysis found that interventions lasting longer than 16 weeks were associated with better outcomes^12^. In contrast, a systematic review did not find a significant association between intervention duration and outcomes^13^. Evidence suggests that physical activity performed 3 times per week at 30 minutes per session would be the recommended dose for patients living with AD, which is already lower than current WHO recommendations for the general population^12^. Among these populations, it may be ineffective to propose targets aimed at healthier and younger populations because efficacy might be reached at a different dose of physical activity. In particular, most trials investigating the effect of physical activity on specific health conditions such as mild cognitive impairment and preclinical AD, are often subject to selection bias that excludes other chronic conditions. Given that the majority of older adults have more than two chronic diseases and over 20% of those aged 75 and above have five or more^14^, this poses a potential impediment to identifying strategies with the greatest real-world effectiveness. Additionally, the lack of sociodemographic representation further contributes to the exclusion of marginalized groups. While group-level studies and implementation science have proven valuable in assessing intervention efficacy and implementation, they often overlook individual factors crucial for sustaining a physically active lifestyle across the lifespan. Considering the complexity and individuality involved in addressing physical inactivity, precision medicine approaches are likely necessary to effectively tackle this global issue.

A precision medicine approach could provide benefits in adherence, targets, and adequate dose response to physical activity interventions. Individual differences in neurobehavioral features, preferences, and barriers (i.e., sociodemographic, cultural, technology proficiency, environmental factors like neighborhood walkability and safety, proximity to green spaces, etc.) can influence intervention adherence and promote voluntary engagement in physically active behaviors. Moreover, the application of brain-derived markers using functional neuroimaging (neural markers), in combination with other modalities (e.g. blood-based biomarkers), has demonstrated their ability to predict future performance in health-related behaviors and response to treatment beyond solely clinical or behavioral variables^15,16^. Other important strides are already underway, for example, Morris et al.^17^ built generalizable models that predicted future adherence or change in behavior. If these models generalize to other populations and outcomes, they can be used to apply to new individuals to predict their future adherence. In terms of dose-response and targets, they can be affected by the type of physical activity (strength, aerobic, walking, cycling, etc.), intensity (High-Intensity Interval Training bouts, light or moderate to vigorous physical activity, etc.), and amount/quantity (e.g., time spent in activity) of physical activity^10^. These adherence and dose-response-associated elements can be based on individual differences in disease state, life stage, and level of risk.

## Existing challenges in physical activity trials

Variability in patient response to treatment poses a challenge for physical activity trials. Even if the intervention is limited to a carefully targeted population, there will be baseline variability in many factors that could influence the outcome of the intervention, such as physical fitness, level of motivation, socioeconomic status, access to greenspace, work/life commitments and other environmental, familial, geographic, and individual differences in social support. Another challenge is ensuring intervention adherence, as it can be difficult to ensure that participants maintain a regular and dedicated commitment to the prescribed program. High dropout rates can also be a challenge, as they can affect the validity of study results and together pose statistical decision-making challenges in terms of treating the analysis as intention-to-treat or per-protocol and average causal complier effects^18^.

Moreover, the lack of generalizability of results is concerning, as volunteer study participants may be highly motivated and not representative of the larger population. Selection bias is a common issue across most clinical research studies where individuals, by simply showing interest in participating in a trial, self-select from the rest of the population. Furthermore, we often recruit from non-representative samples across cultural, linguistic, and sociodemographic characteristics, mainly from Western, educated, industrialized, rich and democratic (WEIRD) societies^19^. This is particularly problematic in behavioral interventions, where sample selection bias might result in healthier and more physically fit participants than the rest of the general population^20^. Finally, longer-term maintenance is one of the most difficult aspects of physical activity interventions. Kaushal et al. (2015)^21^ determined it takes six weeks to develop an exercise habit given that individuals are exercising at least four times per week, and favorable conditions are present to promote automaticity or habit formation. Importantly, though, research suggests that once a successful start is initiated, 60–80% of participants can maintain activity levels at 3-months^22,23^. However, the remaining 40–20% do not. Certainly, patient heterogeneity in baseline characteristics might impact the long-term effects of an intervention, making the maintenance of intervention effects uncertain.

While challenging in group-level efficacy trials, heterogeneity in patient response provides an opportunity for the development of precision approaches. We require variability in baseline characteristics in real-world settings to detect individual features that confer the greatest benefit. However, precision medicine requires large amounts of real-world data, to leverage a personalized data-driven recommendation (Box 1). Producing such recommendations presents considerable challenges such as collecting and integrating quality data from very diverse devices, activity modalities and study designs. These data points likely lay upon a continuum of flexibility ranging from relatively fixed data (environment, socioeconomic status, occupational status) to modifiable data such as brain function, and blood biomarkers to more flexible data like motivation, goals and fitness. Probably the biggest challenge comes when scaling these solutions to very diverse populations across multiple settings, many of which likely would lack the resources to present a larger pool of options.

Box 1 Main challenges in providing precise data-driven recommendations in physical activity

**Table:** 

1. Data collection and integration	• Collecting and integrating high-quality data from various sources such as wearable devices, sensors, and smartphone apps with sufficient temporal precision.
2. Data harmonization	• Ensuring data standardization and quality control.
3. Personalization	• Personalizing physical activity recommendations based on individual characteristics (i.e. health status, fitness level, and goals). • Requires advanced algorithms and machine learning techniques that can capture and amend prescriptions/interventions to changing environmental and internal factors related to an individual’s biodata and personal preferences.
4. Validity of measures	• Accuracy and reliability of physical activity data and recommendations is important to ensure that they are useful and valid.
5. Data privacy and security	• Ensuring the privacy and security of sensitive personal health data.
6. Scalability	• Making precision recommendations widely accessible to people across different groups of older adults and geographic locations requires scalable solutions and technology to adapt to individual characteristics. ○ Settings and locations ○ Disease type and stage
7. Open Science	• Develop infrastructure for data sharing to enhance rigor and discovery.

Box 1. This table describes the challenges that must be addressed to provide accurate and tailored recommendations for physical activity. These challenges require interdisciplinary collaborations between computer scientists, engineers, statisticians, medical professionals, neuroscientists, kinesiologists, psychologists with measurement expertise and other experts to develop effective and reliable solutions for precision physical and reduce selection bias by identifying and adopting evidence-based strategies to recruit diverse participants.

### What makes a successful physical activity intervention?

The goal of precision approaches to physical activity engagement is to identify the right intervention for the right participant at the right time. To better address the challenges faced by behavioral trials targeting physical activity in older adults and adapt the solutions for a precision medicine approach, we need to understand what makes a successful intervention at the level of the individual. The correct combination of factors can lead to the ultimate goal of physical activity interventions and leading a physically active lifestyle across the lifespan which is improved health outcomes, including cardiovascular health, cognitive functions, and quality of life to name a few.

**Fig. 1:**
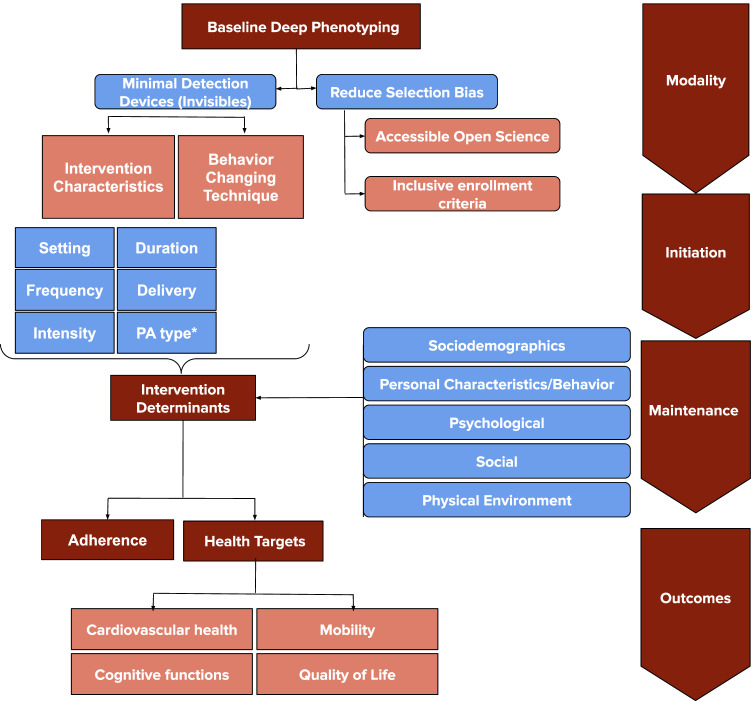
Elements and levels of a behavioral intervention to improve health outcomes and increase adherence to physical activity. *Examples of different types of physical activity (PA) include cardiorespiratory exercise, resistance training, and skill-based activities such as yoga, Tai Chi, and skill-based physical games.

To ensure the effectiveness of a physical activity intervention, it is essential to start by obtaining comprehensive baseline phenotyping (Fig. 1). This approach should be coupled with strategies to reduce selection bias, such as minimizing exclusion criteria and incorporating accessible open data. Additionally, we can enhance measurement techniques to gather high-quality data while minimizing impact, moving from wearables to “invisibles”^24^.

The term “Invisibles” describes an advanced category of wireless and touchless sensors that possess the remarkable ability to capture data without the need for individuals to wear them or provide explicit commands. Invisibles can collect data on movements, activities, and vital signs even across multiple rooms in a smart home by analyzing radio signals. These sensors communicate wirelessly with each other and can be seamlessly integrated with machine learning models to make predictions about individuals’ future health and behavior.

Lastly, it is important to identify individual characteristics that contribute to the likelihood of deriving benefits and maintaining adherence over time. This understanding will enable the personalized prescription of targets and strategies, thereby facilitating the achievement of personalized goals.

A precision approach to physical activity interventions would need to build upon baseline deep phenotyping, reduction of selection bias, and use of multidomain objective measurement of behavior to add another layer of personalization. The proposed framework would include the following elements: (1) determinants of initiation (what is needed to start?), (2) determinants of maintenance (what is needed to sustain engagement?), and (3) patterns of modalities (what type of activity is preferred?). These three elements are influenced by socio-demographics (i.e., age, sex, socio-economic status, ethnicity, etc.), personal characteristics/behavioral (i.e. physical health, mental health, body weight, baseline physical activity, behavior, other lifestyle behaviors, etc.), psychological (i.e. risk perception, self-efficacy, perceived-barriers, perceived benefits, mood, self-motivation, goal setting, etc.), social (i.e. network, support, partner, norms, etc.), and physical environment (i.e. perceived access, traffic and crime safety, street connectivity, etc.) determinants of physical activity among older adults^23^. Successful interventions have managed to assess and target one or more of these determinants in the specific target populations, but a precision approach would extend this to the individual and apply data-driven decisions to empirically test theoretical concepts.

## Emerging opportunities

The advent of digital technologies presents a promising opportunity for developing tailored behavioral interventions targeting physical activity by acquiring health data for deep phenotyping to then provide health information/prescriptions to patients. For instance, the cognitive enhancement field leverages the latest technologies such as augmented and virtual reality, digital forms of meditation, and neurostimulation, to provide personalized and optimized interventions^25^. A closed-loop system with real-time state metrics, real-time data analysis, and adaptive algorithms providing personalized physical activity recommendations would provide a stimulating environment for participating patients. This is the case of interventions like *Just-in-time adaptive interventions* where participants received smartphone-tailored notifications to encourage achieving personalized physical activity goals^26^. While the results in this one feasibility study were modest, baseline deep phenotyping could help improve this approach by matching this intervention to those who are predicted to best benefit from it. To provide personalized recommendations, algorithms require extensive training on the abundance of available data, which is readily accessible through the ubiquity of smartphones. This already existent digital fingerprint needs to be harnessed securely. Addressing data privacy and security challenges is crucial to establishing this framework. As digital technologies advance, they will increasingly facilitate the collection of high-quality data, thereby bringing us closer to realizing this future.

This emerging field can learn from other areas where precision medicine approaches have achieved greater advancements. In cancer, where precision medicine was first applied, there was early integration of the P4 framework: Predictive, Personalized, Preventive, and Participatory medicine^27^. Similar to oncology, the fields of neurology and psychiatry are developing a parallel precision medicine P4 framework, through the lens of an integrative disease modeling approach where systems biology (i.e., genomics, lipidomics, epigenomics, proteomics, metabolomics, transcriptomics, cytomics, microRNAomics, and interactomics) and systems neurophysiology (i.e., structural Magnetic Resonance Imaging [MRI], functional MRI, electroencephalogram [EEG], magnetoencephalography, optical imaging, transcranial magnetic stimulation, metabolic and molecular Positron Emission Tomography, and diffusion tensor imaging) could be combined to produce deep phenotype and profiling of patients across a spectrum of spatial and temporal scales^28^. This approach would allow for the development of patient journey personalization by using predictive biomarkers to inform decisions, provide the right tools for disease prevention, inform patients about treatment options that might be more effective for them to get health benefits, and direct focus on specific patients’ needs^28^.

Similar elements could be incorporated into a proposed Physical Activity Customized Therapy (PACT). Incorporating systems biology and neurophysiology into the behavioral intervention elements from Fig. 1 would allow for patient-deep phenotyping and profiling. Eventually, with enough data, a PACT would incorporate machine learning classification methods so that a given patient would just need to provide basic features to obtain the recommendations most likely to achieve desired health benefits. One example of a successful implementation of this approach is in personalized psychiatry with the development of customized mental health care. However, scaling this approach requires large pools of data on costs and resource utilization (for example, validating brain data using mobile EEG versus MRI) both in the community/at-home-based as well as contextual/intervention based, along with sensitive and ecologically valid outcome measures.

Precision physical activity interventions could be crucial to achieve the next goal of reduction in physical inactivity and to ensure maintenance over time. However, further research and support for open science infrastructure are needed. Using the principles of open science can help harness patient heterogeneity by harmonizing trial data to examine individual baseline differences and also differences in intervention efficacy over time. Current practices already include open-access publishing and pre-registration, but accessible open data in physical activity research is still rare. A start would be to encourage physical activity studies to share their adherence statistics and collection of secondary data. Enhancing infrastructure that supports data sharing and establishing requirements by funding agencies would be crucial in facilitating the application of precision methods to physical activity engagement. The National Institutes of Health (NIH) Data Management and Sharing (DMS) policy is an example of a recent policy aiming to enhance data sharing and open science. It requires funding applicants to plan, budget, manage, and share data, including the submission of a DMS plan for review during the application process. Although in its early stages, if appropriately developed, this collaborative effort has the potential to drive advancements in personalized interventions^29^.

## Building evidence for individualized lifestyle recommendations in the clinic

A PACT involves individualized recommendations to both promote adherence to a given intervention and maximize the health benefits from a given intervention in a given person. The field still lacks sufficient evidence to guide individual-level recommendations. Building that evidence will require diverse study designs from clinical trials with clear standardized endpoints as well as high-quality observational studies that can overcome selection biases and scale to diverse populations. These studies should be accompanied by long-term follow-ups on the effects of these interventions to prevent the development of chronic diseases, delay their onset, and improve health outcomes^30^. Finally, to arrive at a point to be able to provide a targeted recommendation, physical activity trials would need to include standardized outcome measurements (e.g., NIH toolbox, Science of Behavior Change), that can help identify specific amounts and types of physical activity that translate into improved health outcomes.

This roadmap for PACTs has the potential to improve health outcomes and prevent disease by providing individualized physical activity recommendations that are tailored to the unique needs and preferences of individuals. By working with end-users to develop and implement a physical activity plan that is realistic and achievable, PACT could help promote successful aging.

In summary, physical inactivity continues to be a global health problem and a leading risk factor for chronic illness and premature death, particularly among older adults. Despite efforts by public health agencies worldwide to promote physical activity, adherence to recommended guidelines remains low. It is challenging to translate populational level interventions to the individual due to individual differences in participant baseline characteristics and the complexity of physical activity in societies that often promote sedentary behaviors. Precision medicine approaches will harness population heterogeneity in order to guide individualized program development and promote physical activity adherence and ultimately, well-being in aging.
